# Aging effects on neural processing of rhythm and meter

**DOI:** 10.3389/fnagi.2022.848608

**Published:** 2022-09-01

**Authors:** Sarah A. Sauvé, Emily L. W. Bolt, Sylvie Nozaradan, Benjamin Rich Zendel

**Affiliations:** ^1^Faculty of Medicine, Memorial University of Newfoundland, St. John’s, NL, Canada; ^2^Institute of Neuroscience, Université catholique de Louvain, Brussels, Belgium; ^3^Aging Research Centre, Newfoundland and Labrador, Memorial University, Corner Brook, NL, Canada

**Keywords:** aging, frequency tagging, EEG, rhythm perception, beat and meter perception

## Abstract

When listening to musical rhythm, humans can perceive and move to beat-like metrical pulses. Recently, it has been hypothesized that meter perception is related to brain activity responding to the acoustic fluctuation of the rhythmic input, with selective enhancement of the brain response elicited at meter-related frequencies. In the current study, electroencephalography (EEG) was recorded while younger (<35) and older (>60) adults listened to rhythmic patterns presented at two different tempi while intermittently performing a tapping task. Despite significant hearing loss compared to younger adults, older adults showed preserved brain activity to the rhythms. However, age effects were observed in the distribution of amplitude across frequencies. Specifically, in contrast with younger adults, older adults showed relatively larger amplitude at the frequency corresponding to the rate of individual events making up the rhythms as compared to lower meter-related frequencies. This difference is compatible with larger N1-P2 potentials as generally observed in older adults in response to acoustic onsets, irrespective of meter perception. These larger low-level responses to sounds have been linked to processes by which age-related hearing loss would be compensated by cortical sensory mechanisms. Importantly, this low-level effect would be associated here with relatively reduced neural activity at lower frequencies corresponding to higher-level metrical grouping of the acoustic events, as compared to younger adults.

## Introduction

When listening to music, listeners entrain to regularities that are perceived as a musical beat, an integral part of music perception (Huron, 2006). These regularities are usually organized into a meter. A metrical structure consists of a pattern of nested pulse-like beats, which can be but are not necessarily, prominent in the acoustic signal itself (Large, 2008). Increasing evidence suggests that meter perception may be related to selective enhancement of neural activity at the meter periodicities and their harmonics, or meter-related frequencies. This selective enhancement can be observed in the electroencephalography (EEG) signal recorded while listeners are presented with a rhythmic pattern, a series of variable length tones and silences, inducing the perception of a metrical structure (Lenc et al., 2018, 2020, 2021; Nozaradan et al., 2011, 2012, 2016). This selective enhancement has been observed not only in response to regular rhythms with high acoustic energy at the meter frequencies but also in syncopated rhythms, with no prominent acoustic cues to the meter frequencies. These observations suggest that this enhanced neural activity at the meter-related frequencies is not simply stimulus-driven or driven by low-level subcortical processes of the auditory system (Nozaradan et al., 2018a; see also Nozaradan et al., 2016; Lenc et al., 2018, 2020, 2021).

Little is known about the neural processing of rhythmic inputs in older adults. What is known has been focused on speech perception, where overall, the literature suggests that older adults, typically 60+ years, respond more strongly but less precisely to temporally regular stimulation at low (speech-paced) frequencies, approximately 3–4 Hz (Anderson et al., 2012; Bidelman et al., 2014; Goossens et al., 2016; Herrmann et al., 2016; Presacco et al., 2016; Henry et al., 2017), a pattern ascribed to the inhibition theory of aging (Salthouse and Meinz, 1995; Caspary et al., 2008). This theory ascribes an increase in overall brain activity to a decrease in GABA (gamma-aminobutyric acid), an inhibitory neurotransmitter, in the aging brain (Caspary et al., 2008). This decrease in inhibitory activity can also explain the decrease in temporal encoding precision often observed in older compared to younger adults (Anderson et al., 2012; Presacco et al., 2016).

On the other hand, the few pieces of evidence about music perception in older adults suggest that it is generally preserved (Halpern et al., 1995, 1998; Halpern et al., 1996, 2017). In some cases, the performance of a musical task is preserved, but the underlying brain activity changes, suggesting that older adults may automatically compensate for declining cognitive or perceptual abilities in the musical domain (Halpern et al., 2017; Lagrois et al., 2018). This may be due to the development of compensatory mechanisms similar to those found for speech in noise perception, where older adults use contextual information to help overcome age-related changes in hearing and cognitive abilities (Horn and Cattell, 1967; Horn, 1982; Madden, 1992; Birren and Fisher, 1995; Pichora-Fuller et al., 1995). For example, when rating the fit of a note in a melody, older adults relied more heavily on scale degree information, derived from knowledge of the tonal hierarchy, whereas younger adults relied more heavily on note-to-note information (Bharucha and Krumhansl, 1983; Halpern et al., 2017). In addition to increased use of contextual and crystallized knowledge, the neural compensation theory of aging stems from the observation of patterns of over- and under-activation, reduced asymmetry and reduced connectivity of brain activity in the brains of older adults as compared to younger adults when performing a similar task at a similar level (Reuter-Lorenz et al., 2000; Cabeza, 2002; Reuter-Lorenz, 2002; Grady et al., 2003; Cabeza et al., 2004; Reuter-Lorenz and Cappell, 2008; Davis et al., 2011). For example, a frontal shift in activity has been observed in older adults associated with compensation for declining perceptual abilities (Park and Reuter-Lorenz, 2009).

In terms of rhythm perception, Sauvé et al. (2019) used frequency tagging to compare neural activity in older and younger adults while passively listening to a metronome. This study found that older adults, compared to younger adults, had reduced amplitude of neural activity at the frequency of the metronome and to a lesser extent also at the first three harmonics of the metronome frequency (Sauvé et al., 2019), which could not be fully accounted for by age-related changes in hearing as measured by pure-tone thresholds. In other words, older adults demonstrated a reduced response to the metronome frequency compared to younger adults, a pattern in line with literature suggesting that temporal precision degrades in the aging brain (Anderson et al., 2012; Herrmann et al., 2019).

The goal of the current study was to explore differences in neural processing of rhythm between older adults and younger adults actively listening to two rhythms. One rhythm contained prominent acoustic energy at the meter-related frequencies compared to meter-unrelated frequencies (i.e., non-syncopated rhythm). In contrast, the second rhythm was characterized by a lack of prominent acoustic energy at meter-related frequencies compared to meter-unrelated frequencies (i.e., syncopated rhythm; see Lenc et al., 2018, 2020). In the syncopated rhythm, perception of the meter thus likely relies on endogenous processes, and selective enhancement of the neural activity at meter-related frequencies cannot be explained by acoustic confound or subcortical processing enhancing prominent acoustic features of the input (Lenc et al., 2020). To the best of our knowledge, the current study is thus the first to explore the impact of aging on neural processing of rhythm, especially when the meter is inferred but not conveyed by a strictly (i.e., metronomic) or strongly periodic (i.e., non-syncopated) stimulus.

Based on our previous findings (Sauvé et al., 2019), together with the fact that older adults have increased exposure to musical patterns due to their age, and that older adults rely on internalized musical structure as a way to compensate for age-related hearing impairment as compared to younger adults (Halpern et al., 2017), it is expected that older adults will show similar neural activity to younger adults in response to both rhythms despite age-related hearing loss, thus with preserved selective enhancement at the meter-related frequencies irrespective of rhythm complexity. Furthermore, one of the hallmarks of aging is that older adults tend to have more difficulty processing stimuli presented at a faster rate (Salthouse and Meinz, 1995). It is therefore possible that differences between older and younger adults are more pronounced at a higher tempo. Finally, given known topographic neural shifts associated with aging (e.g., Park and Reuter-Lorenz, 2009), we also examined possible differences in topographic maps between older and younger adults. We expected to find selective enhancement at meter-related frequencies over a larger surface for older adults than for younger adults.

## Materials and methods

### Participants

Twenty-nine participants (the same as in Sauvé et al., 2019) took part in this study and provided written informed consent in accordance with the Interdisciplinary Committee on Ethics in Human Research at Memorial University of Newfoundland. Participants were divided into two *age groups*: 15 older adults (>60 years; 10 female) and 14 younger adults (<25 years; 7 female). All participants self-reported being healthy, right-handed and free of any cognitive deficit. All participants were non-musicians, although some had music training in childhood (specifically, 8 younger and 2 older adults). Hearing abilities were assessed using standard clinical pure-tone (PT) audiometry, which revealed significant differences between the two age groups, as detailed in Table 1. There was no difference in hearing thresholds between participant who had received some musical training and those who had received none [*t* (28) = −1.84, *p* > 0.05]. All participants received a small cash honorarium for their participation. Table 1 summarizes participant demographics.

**TABLE 1:** Participant demographics.

	Age	Formal education (years)^a^	Pure-tone average (dB HL)^b^
Younger adults	20.3 (1.84)	14.6 (2.23)	6.2 (5.07)
Older adults	63.4 (3.68)	12.87 (3.46)	13.28 (9.03)

^a^*t*(28) = 1.6, *p* ≥ 0.05; standard deviation in brackets.

^b^Binaural average of pure-tone threshold at 500, 1,000, and 2000; *t*(28) = 40.55, *p* < 0.05; standard deviation in brackets.

### Stimuli

**FIGURE 1:**
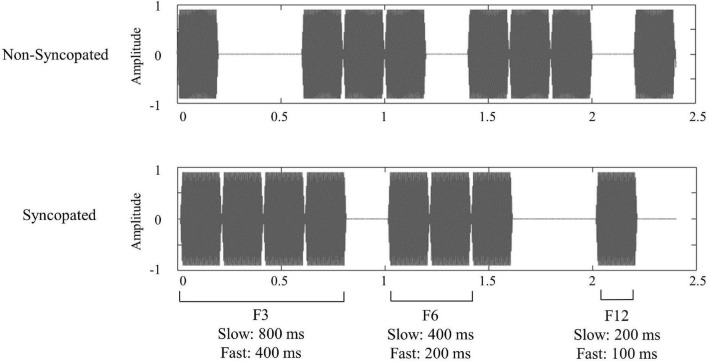
Audio signal representation of the non-syncopated and syncopated rhythms at slow tempo (with 200 ms individual sounds).

The selected rhythmic patterns were similar to those of previous studies (Nozaradan et al., 2016, 2017, 2018b; see also Lenc et al., 2018) and presented into four *conditions*: two *rhythms* (*non-syncopated* and *syncopated*) at two *tempi* (*slow*: 0.416 Hz cycle rate, i.e., rhythmic pattern of 2,400 ms duration; and *fast*: 0.833 Hz cycle rate, i.e., 1,200 ms pattern duration). The structure of the rhythms was based on the alternation of 12 events, consisting of sounds and silent intervals (Figure 1). The individual sounds consisted of 1,000 Hz pure tones, presented at ∼75 dB SPL, with 10 ms rise and fall times, and were either 100 or 200 ms long for the fast or slow tempo, respectively. All stimuli were presented via insert earphones (Etymotic E3A). As described in Nozaradan et al. (2016) and Lenc et al. (2018), rhythms were designed to induce the perception of a meter based on a preference for grouping by 2 and 4 events per cycle (grouping by 4 events corresponding to the periodicity mostly tapped by participants when asked to tap on the rhythm; see Lenc et al., 2018). The first rhythm could be considered non-syncopated, as the rate of the perceived meter levels closely matched with the groups of acoustic events making up the rhythm. This matching was corroborated by frequency-domain analysis of the envelope of acoustic signal, confirming prominent acoustic energy at the meter-related frequencies (see also Lenc et al., 2021 for complementary measures of syncopation on the same rhythm). In contrast with the first rhythm, the second rhythm was considered syncopated, as the rate of the perceived meter levels did not closely aligned with the groups of sounds making up the rhythm, due to a more irregular arrangement of groups of tones and silent intervals making up the rhythm. This was also reflected in frequency-domain analysis of the envelope of the acoustic stimulus, showing that the meter-related frequencies were not salient in the stimulus (Lenc et al., 2021). Stimuli were generated in Reaper (V 5.16) and presented to participants using Eprime (V 3.0).

### Procedure

After providing written informed consent, participants completed a short demographics questionnaire and were given the possibility to practice tapping to the 4 rhythms that would be presented during the study. Each participant was prepared for EEG recording and was seated in a double-walled, electrically shielded sound-attenuating booth (Eckel). Participants were given a response box (Chronos, EPrime) and could see a computer monitor. They were further instructed to tap their index finger with their dominant hand in time with the perceived beat while listening to the stimuli. They tapped on one of the response buttons whenever the green light came up on the response box, and the computer monitor said, “Tap.” When the response box buttons became red and the computer monitor said “Stop Tapping,” participants were instructed to stop tapping but to maintain the beat mentally while the stimuli continued playing. Each participant was told that they would be expected to start tapping as soon as the green light came back on. Alternating between a Tapping Phase and a Listening Phase was done to ensure that participants actively maintained the meter throughout the task. To avoid eye movement, participants were told to either fixate on the monitor or response box during the task and to minimize switching between the two.

The two rhythms (non-syncopated, syncopated) were presented at the two tempi (slow, fast) in four separate blocks. The order of the blocks was identical for each participant (non-syncopated rhythm, slow and fast, and then syncopated rhythm, slow and fast). This fixed order was used to ensure that participants could easily tap the perceived meter during the tapping phase and were thus likely to perceive the meter also when listening without tapping. Indeed, pilot testing suggested that participants had increased difficulty tapping to the fast vs. slow stimuli, and to the syncopated vs. the non-syncopated rhythm, even after a short practice session. In contrast, the participants seemed overall more comfortable tapping when they were first presented with the non-syncopated rhythms and the slow tempi. Each block started with a Tapping Phase that lasted 48 s. After this Tapping Phase, there were 10 repetitions of a 48 s Listening Phase and a 12 s Tapping Phase.

### Electroencephalography data processing

Neuroelectric brain activity was digitized at a sampling rate of 1024 Hz from 134 electrodes using the radial layout system, with a highpass filter set at 0.1 Hz using a Biosemi ActiveTwo system (Biosemi Inc., Amsterdam, Netherlands). Six electrodes were placed bilaterally at mastoid, inferior ocular and lateral ocular sites.

Data wereanalysed using the Letswave 6^1^ (Mouraux and Iannetti, 2008) signal processing toolbox for Matlab. Eye blinks and eye movements were identified for each participant using an independent component analysis (ICA). Components related to eye movements were removed from the EEG signal. Next, the EEG data were referenced to the average of all scalp electrodes. Each condition was divided into ten 48 s epochs corresponding to the Listening Phases and then averaged into a single 48 s epoch, yielding four epochs (*non-syncopated slow*, *syncopated slow, non-syncopated fast, syncopated fast*) per channel and participant. EEG data recording during the tapping phase was not used for further analysis, as the purpose of the current study was not to focus on the neural activity related to overt rhythmic movement production. However, this limited tapping data can provide a behavioral index about the perceived meter using inter-tap interval (ITI) information (see Supplementary material).

To measure the amplitude at the meter-related and meter-unrelated frequencies elicited in response to the rhythms, a fast Fourier transform (FFT) was applied to the four EEG epochs for each EEG channel and participant, with a resulting spectral resolution of approximately 0.02 Hz (i.e., 1/48 s). These spectra were then corrected using a noise subtraction with bin range (±) 3–13 (0.2 Hz on each side beginning at 0.06 Hz). This function, which subtracts from the amplitude at each frequency bin the amplitude averaged across the neighboring frequency bins from the 3rd to the 13th bin on each side (Mouraux et al., 2011; Nozaradan, 2013; Nozaradan et al., 2015), thus centers the amplitude spectrum around zero. Therefore, the amplitude measured at the target frequencies should tend toward zero if no significant neural activity is elicited at those specific frequencies. The amplitude of neural activity obtained from this analysis will be referred to as simply *amplitude* throughout this paper.

**FIGURE 2:**
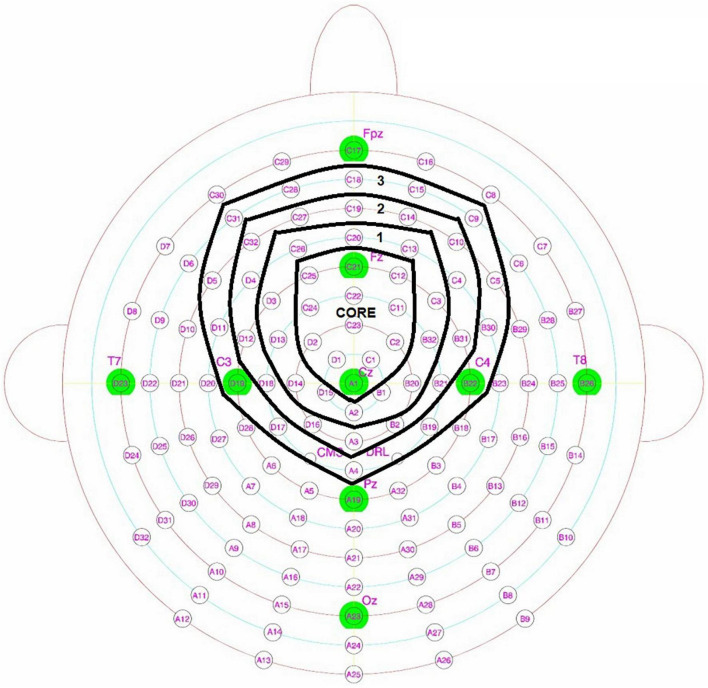
128 + 2 radial electrode layout illustrating core and outer circle electrode montages.

For each *condition*, the amplitudes at targeted frequency bins were extracted from a montage of frontocentral electrodes in increasingly large concentric circles (see Figure 2). The *core circle montage* was selected based on signal distribution and consistent with previous work (Nozaradan et al., 2012, 2016; Lenc et al., 2018), while each subsequent *outer circle* includes one electrode in each direction from the core. This analysis was done to investigate any potential topographical differences between age groups. Previous work has suggested a compensation theory of aging (Reuter-Lorenz and Cappell, 2008), which predicts larger responses or more widespread activation in older adults. Given that the magnitude of the response and the extent of the spatial distribution are difficult to separate, it is important to test both possible effects by comparing both the magnitude of the responses and their spatial spread. By testing topographic patterns as a function of age, the possibility of age-related differences during meter perception tasks can be explored.

As in Lenc et al. (2018), the amplitude at 12 target frequencies was extracted, corresponding to the duration of the whole pattern/cycle rate (0.416 or 0.833 Hz for the two different tempi, respectively) and its harmonics up to the period of single acoustic events (5; 10 Hz). These 12 frequencies were then tagged as meter-related frequencies and meter-unrelated frequencies (Lenc et al., 2018). The meter-related frequencies were based on the periodicities of the meter assumed to be perceived for these rhythms and their harmonics, as informed by behavioral data collected in a large number of participants in previous studies (Nozaradan et al., 2016, 2018b; Lenc et al., 2018) and corroborated by the tapping data collected during the Tapping Phases. These meter periodicities consisted in the smallest inter-sound intervals (100 and 200 ms for the fast and slow tempi respectively), and grouping of these intervals by 2, 4, and 12 intervals (see Lenc et al., 2018, for example), thus yielding four meter-related frequencies (i.e., the 1st, 3rd, 6th, and 12th frequencies from the 12 target frequencies, further labeled F1, F3, F6, and F12). The remaining frequencies were considered meter-unrelated. Amplitudes were obtained for each frequency of interest from the closest spectral bin from each frequency.

### Statistical analysis

Statistical analyses were conducted using mixed effects multiple linear regression modeling implemented in the R (3.3.2) *lme4* (Bates et al., 2015) package. All analysis models were maximally fitted to reflect the experimental design, as per Barr et al. (2013), where models included random intercepts on participants and random slopes on participants by stimulus condition. All categorical variables were factors, where each level was compared to a base level. These base levels are *slow* for *tempo*, *non-syncopated* for *rhythm*, *younger* for *age group*, *meter-related* for *frequency type, F3* for *frequency, no training* for *musicianship* and *core* for *electrode montage*. Note that the choice of base level does not affect the overall results of the model, only the direction and magnitude of the coefficients. For example, mean frequency amplitudes remain constant; reporting differences between each frequency and F3 would result in different numbers than reporting differences between each frequency and F6. Models were evaluated using Pearson’s correlation between the model’s predictions and the data along with the correlation’s 95% CIs. Statistical significance of each individual factor level for a given predictor was evaluated using 95% CIs, where an interval not including zero indicates a significant predictor. In each analysis, follow-up independent sample two-tailed *t*-tests with Bonferroni correction were conducted to investigate simple effects of age. Alpha was 0.05.

#### Frequency analysis

For each participant and condition, we first averaged the amplitudes for meter-related frequencies and meter-unrelated frequencies separately. Then, we compared the amplitude of the response at meter-related and meter-unrelated frequencies to measure selective enhancement of brain activity at meter-related frequencies across stimuli. This is especially important in the case of the syncopated rhythm, which does not include prominent meter-related frequencies. To do so, a linear mixed effects model predicting spectral amplitude of neural activity included *frequency type* (*meter-related, meter-unrelated*), *tempo* (*slow*, *fast*), *rhythm* (*non-syncopated, syncopated*), *musicianship (no training, training)*, and *age group* (*younger, older*) as fixed effects including interactions and *PTAv* (pure tone threshold average) score as a fixed main effect. PTAv was calculated by taking the binaural average of pure-tone threshold at 500, 1,000, and 2,000 Hz and was used to check for low-level effects of poorer hearing in older adults while channel accounts for variance between electrodes. As expected, the PTAv was larger in older adults compared to younger adults, demonstrating normative age-related changes in hearing thresholds (see Table 1).

Next, to investigate age effects on meter-related entrainment, a model predicting spectral amplitude of neural activity in response to meter-related frequencies included *tempo* (*slow, fast), rhythm (non-syncopated, syncopated), frequencies* (*F1, F3, F6, F12*), *age group* (*younger, older*), and *PTAv* score as fixed effects. In all models, interactions with PTAv were not modeled. Finally, the neural compensation hypothesis predicts more widespread activation in older adults than in younger adults (Reuter-Lorenz and Cappell, 2008). To test this, *montage (core, outer 1, outer 2, outer 3)* was added to the meter-related entrainment model as a fixed effect, including interactions (except with PTAv).

#### Tapping analysis

The tapping task was included to ensure the participant was focusing on the metrical structure while further listening without moving. The tapping data were also used as a behavioral index confirming the assumed perceived metrical structure. As expected, participants were mostly tapping at intervals aligning with grouping by two, four, or eight intervals, with the majority converging on groupings of 4 (see Supplementary material 2). Mean (standard deviation) ITIs for slow non-syncopated, fast non-syncopated, slow syncopated and fast syncopated conditions were 781.1 (186.1), 400.34 (97.2), 791.4 (193.2), and 434.1 (135.7), respectively, where 800 and 400 ms represent groupings of 4 for the slow and fast conditions, respectively. Because only a small number of taps were recorded between each rest period and because a proper analysis of the precision of tapping, for example, requires a significantly higher number of taps (i.e., >100 in Zendel et al., 2011), the tapping data were not used for further exploration of the tapping performance. Instead, the data are presented with a cursory analysis of mean inter-tap interval as Supplementary material 2.

## Results

### Meter-related vs. meter-unrelated frequencies

**FIGURE 3:**
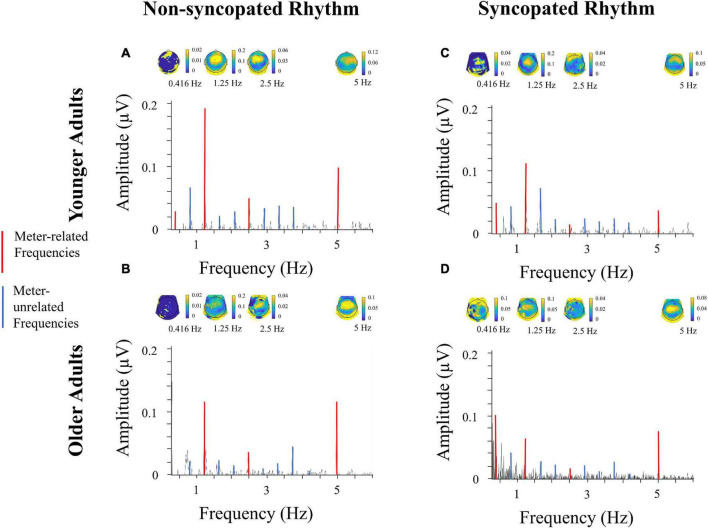
EEG spectra, slow tempo. Amplitude of neural activity at electrode Cz ranging from 0.5 to 6 Hz for *younger adults* **(A,C)** and *older adults* **(B,D)** adults for *non-syncopated* **(A,B)** and *syncopated* **(C,D)** rhythms for *slow tempo*, along with topographic maps corresponding to each meter-related frequency. Frequency and amplitude scales are identical across conditions and groups to allow direct comparison across spectra. The scales of the topographical maps are adjusted for each condition, group and frequency for optimal visualization of topographical distribution across the EEG channels. Each topographical map corresponds to a meter-related frequency, shown in red.

**FIGURE 4:**
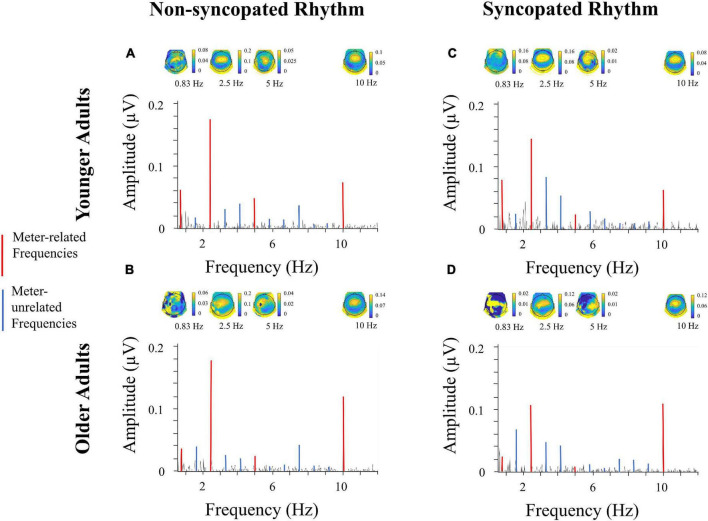
Electroencephalography (EEG) spectra, fast tempo. Amplitude of neural activity at electrode Cz ranging from 0.9Hz to 12Hz for *younger adults* **(A,C)** and *older adults* **(B,D)** for *non-syncopated* **(A,B)** and *syncopated* **(C,D)** rhythms for *fast tempo*, along with topographic maps corresponding to each meter frequency. Frequency and amplitude scales are identical across conditions and groups to allow direct comparison across spectra. The scales of the topographical maps are adjusted for each condition, group and frequency for optimal visualization of topographical distribution across the EEG channels. Each topographical map corresponds to a meter-related frequency, shown in red.

**FIGURE 5:**
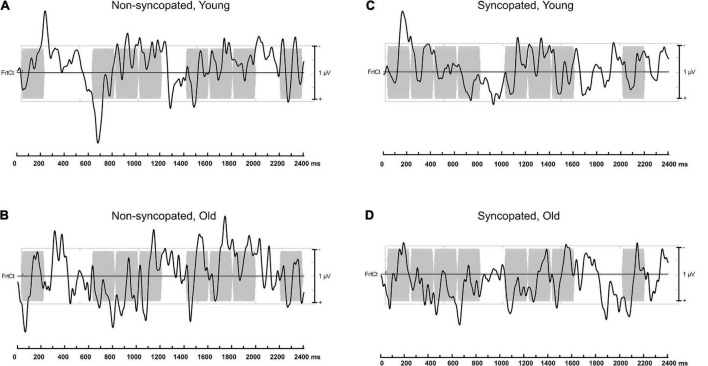
Electroencephalography (EEG) data presented in the time-frequency domain averaged across each repetition of the rhythm and the montage of fronto-central electrodes used in analysis for *younger adults* **(A,C)** and *older adults* **(B,D)** for *non-syncopated* **(A,B)** and *syncopated* **(C,D)** rhythms for *slow tempo* overlaid onto their respective auditory stimuli.

**FIGURE 6:**
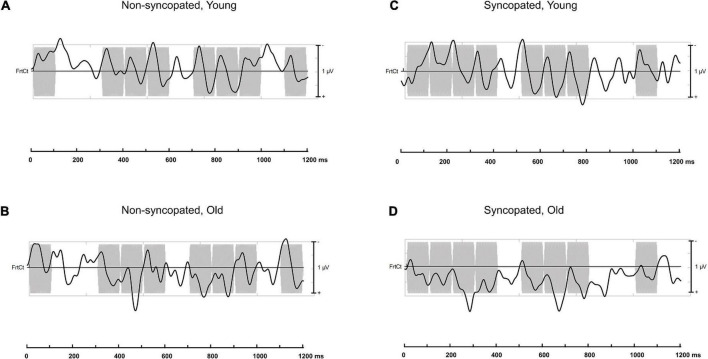
Electroencephalography (EEG) data presented in the time-frequency domain averaged across each repetition of the rhythm and the montage of fronto-central electrodes used in analysis for *younger adults* **(A,C)** and *older adults* **(B,D)** for *non-syncopated* **(A,B)** and *syncopated* **(C,D)** rhythms for *fast tempo* overlaid onto their respective auditory stimuli.

First, we tested for selective enhancement of brain activity elicited at frequencies corresponding to the meter by comparing amplitudes at meter-related frequencies vs. meter-unrelated frequencies using a linear mixed effects model. Figures 3, 4 present the brain responses along with topographic maps for each meter-related frequency for the *slow* and *fast* tempi, respectively. Figures 5, 6 present the time-domain EEG response overlaid with the acoustic stimulus. The model’s predictions were significantly correlated with the data, *r* = 0.39, 95% CIs [0.38,0.40], *p* < 0.01; full model details can be found in Supplementary material 1. Main effects of *tempo* (*slow* > *fast), rhythm* (*non-syncopated* > *syncopated), frequency type* (*meter-related* > *meter-unrelated)*, and *musicianship (training* > *no training)* were significant while *age group (younger* > *older)* and *PTAv* were not. Only *intercept* and *frequency type* had R^2^ values over 0.01,0.07, and 0.10, respectively, indicating that the random effects and frequency type had the most explanatory power of all the predictors. Significant interactions include all two-way interactions except for between *rhythm* and *tempo*, *frequency type* and *age*, and *tempo* and *age*, three-way interactions between *age*, *rhythm* and *frequency type*, *age*, *tempo*, and *rhythm, musicianship, rhythm* and *frequency type, musicianship, tempo* and *frequency type, musicianship, age* and *frequency type*, and four-way interactions between *age, tempo, rhythm* and *frequency type*, and *musicianship, age, tempo* and *frequency type*. These interactions were driven by the size of the effect of frequency type, musicianship and age.

**FIGURE 7:**
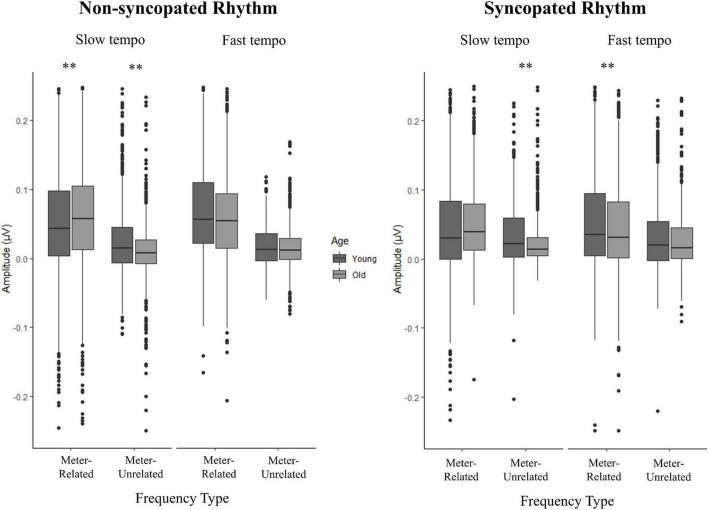
Amplitude of neural activity at meter-related and meter-unrelated frequencies across the four conditions and groups. Boxes represent the first quartile, median and third quartile of data, vertical lines the maximum and minimum values as defined by third and first quartile ± 1.5 times the interquartile-range (third quartile - first quartile), with points representing outliers; data represents amplitude values for each frequency (4 m-related and 8 m-unrelated frequencies), channel (12 channels) and participant, totaling approximately 720 and 1440 data points for meter-related and meter-unrelated frequency types, respectively. **p < 0.006 (Bonferroni corrected).

In the case of frequency type, the effect was similar for the *fast syncopated*, *slow non-syncopated* and *fast syncopated* conditions [*d* = 0.57, *t* (1865) = 14.66, *p* < 0.012 (Bonferroni corrected), *d* = 0.59, *t* (1727) = 14.47, *p* < 0.012 and *d* = 0.59, *t* (2474) = 15.88, *p* < 0.012 respectively], and larger for the *fast non-syncopated* condition [*d* = 0.97, *t* (1523) = 24.86, *p* < 0.012]. Musicianship had an effect for young adults at the slow tempo for both rhythms and frequency types, as well as in the fast syncopated condition for meter-related frequencies (see Table 2). Musicianship had an effect for older adults in the slow syncopated condition for meter-unrelated frequencies and in the fast non-syncopated condition for meter-unrelated frequencies (see Table 2). For younger adults, musicianship led to higher amplitudes; for older adults, to lower amplitudes. See Supplementary material 1 for details of all musicianship follow-up tests. In the case of age, Figure 7 illustrates the size of the effects. To summarize, in the slow conditions for meter-related frequencies, older adults had higher amplitudes than younger adults. In the slow conditions for meter-unrelated frequencies, older adults had smaller amplitudes than younger adults. Age differences were smallest in the fast conditions. Not all of these age comparisons were significant (see Figure 7). There was no main effect of age, but there were some interactions involving age, showing that selective enhancement of meter frequencies was larger in younger adults in some conditions but not all conditions (e.g., meter-related frequencies at slow tempo). A more detailed analysis of meter-related frequencies (section “Amplitude across meter-related frequencies”) explores age differences further. Figure 7 illustrates the mean amplitude of neural activity for all meter-related and meter-unrelated frequencies and all four conditions for older and younger participants.

**TABLE 2:** Summary of significant simple musicianship effects for frequency type analysis, including tempo, rhythm, frequency type, and age.

Tempo	Rhythm	Frequency type	Age	Statistic	Degrees of freedom	Effect size
Slow	Non-syncopated	Meter-related	Younger	−3.30	655	−0.25
		Meter-unrelated	Younger	−3.42	1,260	−0.18
	Syncopated	Meter-related	Younger	−9.69	644	−0.70
		Meter−unrelated	Younger	−4.18	1,301	−0.23
			Older	6.20	371	0.34
Fast	Non-syncopated	Meter-unrelated	Older	4.85	285	0.32
	Syncopated	Meter-related	Younger	−6.38	571	−0.48

The *t*-statistic along with degrees of freedom and Cohen’s *d* are reported.

### Amplitude across meter-related frequencies

**FIGURE 8:**
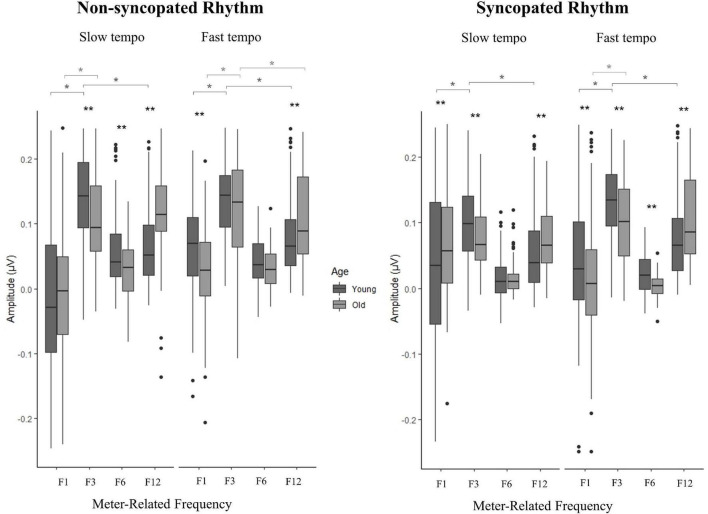
Amplitude of neural activity at the different meter-related frequencies across the four conditions. Boxes represent the first quartile, median and third quartile of data, with points representing outliers; data represents amplitude values for each frequency (4 m-related frequencies), channel (12 channels) and participant. **p < 0.003 (Bonferroni corrected) for all age comparisons. *p < 0.006 (Bonferroni corrected) for all frequency comparisons.

Next, we analyzed the amplitudes across the meter-related frequencies to determine whether the pattern of distribution of the amplitudes was affected by age (Sauvé et al., 2019). Table 3 presents the linear mixed effects model for the amplitude of neural activity elicited at meter-related frequencies for all participants across conditions. For brevity, only significant predictors with R^2^ above 0.01 were included in Table 3; full tables can be found in Supplementary material 1 along with corresponding summary statistics. Significant predictors included main effects of *rhythm* (*non-syncopated* > *syncopated*), *age group* (*younger* > *older*), and *frequency* (*F1* < *F3* > *F6* < *F12, or F1* < *F6* < *F12* < *F3*) as well as multiple two-, three- and four-way interactions. To further understand the interactions of *age group* with *tempo*, *rhythm* and *frequency*, follow-up t-tests were conducted (see Supplementary material 1). Age effects were observed at F3 in all but one tempo and rhythm condition (slow syncopated), where younger adults had larger amplitudes than older adults (Figure 8, *p* < 0.003). Younger adults also had significantly larger amplitudes than older adults at F6 for the slow non-syncopated and fast syncopated conditions, while age groups had similar amplitudes at F6 for the fast non-syncopated and slow syncopated conditions. An age effect was significant at F1 in all but one condition (slow non-syncopated). At F1, younger adults had larger amplitudes than older adults in the fast non-syncopated and syncopated conditions while older adults had larger amplitudes than younger adults in the slow non-syncopated and syncopated conditions. This opposite age effect, where older adults had larger amplitudes than younger adults, was observed at F12 in all conditions but was not significant in the slow syncopated condition. In summary, younger adults had larger amplitudes than older adults at F1 in the fast tempo condition, and F3 and F6 at both tempi. Older adults had larger amplitudes than younger adults at F1 in the slow tempo condition and at F12 for both tempi. Figure 8 presents the mean amplitude for younger and older adults as a function of rhythm, tempo, and frequency.

**TABLE 3:** Summary of significant predictors with *R*^2^ > 0.01 from the maximally fitted multiple linear regression model predicting neural activity amplitude at meter-related frequencies, including coefficient, Wald 95% confidence intervals and *R*^2^ for each predictor (where there are multiple levels, *R*^2^ is given for the predictor as a whole).

Predictor	Coefficient	2.5%	97.5%	*R* ^2^
Intercept	0.22	0.19	0.24	0.10
F1	−0.22	−0.24	−0.20	0.20
F6	−0.15	−0.17	−0.14	
F12	−0.13	−0.15	−0.11	
Age:F1	0.06	0.03	0.08	0.01
Age:F6	0.06	0.03	0.08	
Age:F12	0.12	0.10	0.15	
Rhythm:F1	0.17	0.14	0.19	0.03
Rhythm:F6	0.06	0.04	0.09	
Rhythm:F12	0.10	0.07	0.12	
Tempo:Rhythm:F1	−0.14	−0.18	−0.11	0.01
Tempo:Rhythm:F6	−0.06	−0.10	−0.03	
Tempo:Rhythm:F9	−0.09	−0.12	−0.05	

Random effects account for <0.01 variance and are therefore not reported.

*r* = 0.64, 95% CIs [0.63, 0.66], *p* < 0.01.

Finally, in order to further characterize the amplitude profiles, *t*-tests with Bonferroni correction were conducted between F3 and F1 and F3 and F12 amplitudes for each age group and condition (see Figure 8 and Supplementary material 1 for details). F3 had larger neural amplitude than F1 for both age groups in all conditions except for older adults in the slow syncopated condition (*p* < 0.006). The F3 was larger than the F12 in younger adults, while this difference was not significant in older adults, except during the fast non-syncopated condition.

### Neural compensation

Finally, we investigated the distribution of the signal at meter-related frequencies to test the hypothesis that older adults demonstrated more widespread neural activation than younger adults. First, the main effect of *montage* was significant, where overall mean amplitude decreased with increasing distance from the *core montage*. This main effect was qualified by a two-way interaction with *frequency*, and a three-way interactions with *age* and *frequency*, and *tempo* and *frequency*. The three-way interaction involving age is driven by the difference between F3 and F12, where younger adults have larger amplitudes than older adults at F3 and vice versa for F12. In terms of frequency, the overall pattern was similar across electrode montages except for F1, where the amplitude of F1 diminished faster than the other target frequencies as distance from the core increased. In summary, we did not find any evidence of more widespread neural activation in older adults as compared to younger adults.

## Discussion

In this study, we examined differences between older and younger adults in neural activity to a syncopated and a non-syncopated rhythm presented at two different tempi. Overall, the amplitude of neural activity elicited at meter-related frequencies was larger compared to the activity elicited at frequencies unrelated to meter periodicities, even in the syncopated rhythm where the meter-related frequencies are not prominent acoustic features of the input. This finding is consistent with previous work (Nozaradan et al., 2016, 2018b; Lenc et al., 2018, 2020). Most importantly for our experiment, this effect was similar across older and younger adults, thus suggesting that the processes by which the meter-related frequencies are selectively enhanced is generally preserved in aging, in some conditions diminished and in others augmented, despite hearing loss. Moreover, the difference in pure tone audiometric thresholds between age groups did not have a significant impact on the amplitudes of neural activity. Therefore, differences in neural activity observed here between age groups are unlikely to be fully attributable to low-level hearing abilities. It is worth noting that the method used here cannot dissociate whether a difference in z-score values of meter-related frequencies across group is due to differences in the magnitude of neural responses, their timing, or both (Rajendran et al., 2017). However, the fact that the method is sensitive to both differences in amplitude and differences in phase stability at the tagged frequencies over the long trials precisely makes it a highly sensitive measure to capture any difference in selective enhancement of meter-related frequencies. Such a high sensitivity is critical for comparison of EEG responses across groups. Notably, despite this high sensitivity, no significant difference was observed across age groups. This null result suggests that older participants showed fairly preserved neural responses to acoustic rhythms despite significant peripheral hearing loss. Interestingly, the experience of musical training had opposing effects on the age groups. For younger adults, some musical training is related to higher amplitudes; for older adults, to lower amplitudes, though there was rarely a significant difference for older adults. This may be due to the recency, or lack thereof, of said musical training. In other words, childhood musical training for younger adults is more recent than childhood musical training for older adults. Our results suggest that the benefit of musical training disappears after a certain amount of time without training. Future studies collecting more detailed information about musical experience can further investigate this effect.

Though both age groups show selective enhancement at the meter periodicities, the distribution of amplitudes across the different meter-related frequencies differs between age groups. In older adults, we observe lower amplitude at F3 across stimulus conditions compared to younger adults, consistent with an overall decline in delta band frequency activity in normal aging (Polich, 1997). This age effect is in line with the findings of Sauvé et al. (2019), where the same participants were listening to an isochronous rhythm at 1.25 and 2.5 Hz and where older adults had lower amplitudes at the stimulus fundamental frequency (analogous to F3 in this study) than younger adults. In contrast, this effect does not directly fit with the speech neural entrainment literature which shows increased amplitude of neural activity at low (syllable-paced) frequencies of 3–4 Hz in older adults (Goossens et al., 2016; Herrmann et al., 2016, 2019). One case where younger adults showed higher neural amplitude in response to a 2.8 Hz frequency-modulated stimulus than older adults involved attentional control: younger adults demonstrated more activity at 2.8 Hz in an active task when compared to as passive task, where older adults had no difference and had activity similar to younger adults during the passive task (Henry et al., 2017). However, in Sauvé et al. (2019), the task was passive, so attentional control cannot explain younger adults’ larger amplitudes as compared to younger adults. Furthermore, these studies only investigated neural responses at the fundamental frequency corresponding to the periodicity of the isochronous sequence, while Sauvé et al. (2019) also included harmonics of that frequency, observing a different pattern of distribution of amplitude across the fundamental frequency of stimulation and its harmonics in older adults compared to younger adults.

In the current study, younger adults demonstrate larger amplitude at F3 compared to F12 in the two rhythms and tempi, in contrast with older adults who do not show such distribution across meter-related frequencies. Sauvé et al. (2019) also observed an overall more equally distributed amplitude across the fundamental frequency and harmonics in older vs. younger adults in the EEG response to an isochronous sound at 1.25 and 2.5 Hz. Since the difference is not modulated by rhythmic complexity, it could be explained by an aspecific change in low-level response to auditory rhythmic inputs, irrespective of meter processing. This is compatible with previous observation of larger N1-P2 evoked potentials elicited in response to auditory stimuli in older adults than younger adults (Anderer et al., 1996; Alain and Woods, 1999; Ross et al., 2009; Zendel and Alain, 2014). As Zendel and Alain (2014, p. 61) explain, “This pattern of results has been attributed to a failure to inhibit irrelevant auditory information as a result of age-related changes in prefrontal functions (Knight et al., 1999; Kok, 1999; Bertoli and Probst, 2005). Evidence for this hypothesis comes from neurological studies showing that patients with lesions in the dorsolateral prefrontal cortex have an enhanced P1 (Alho et al., 1994) and N1 (Knight et al., 1980; Woods and Knight, 1986) amplitude relative to age-matched controls. Hence, the dorsolateral prefrontal cortex appears to play an important role in gating sensory input to the auditory cortex, although the exact mechanisms by which this is achieved remain to be determined. Moreover, the age-related difference in P1 and N1 amplitude was smaller during active compared to passive listening, which suggests that focused attention may mitigate some of the age-related changes in inhibitory function. Finally, it is possible that age-related decline in N2 amplitude was also related to deficits in inhibitory functions (Čeponienė et al., 2008).” This low-level sensory effect has been linked to reduced adaptation recovery times underlying temporal processing of tone sequences (Herrmann et al., 2016). In the context of this study, it is important to note that the frequencies corresponding to individual acoustic events used to make up the inputs (i.e., frequency F12, corresponding to 200 and 100 ms in slow and fast tempo, respectively) are congruent with the temporal course of typical N1-P2 potentials. Therefore, based on the evidence that older adults generally demonstrate larger N1-P2 amplitudes, it would be expected to find proportionally larger amplitudes at F12 compared to the slower frequency F3 in older adults compared to younger adults, regardless of rhythm complexity and tempo. The approximate wavelength of the N1-P2 complex is 100–200 ms, yielding a frequency of 10 and 5 Hz, respectively. It is therefore possible that the enhancement at these frequencies observed in older adults maps on to the enhanced N1-P2 complex that is typically observed in older adults. In other words, this effect is not related to metrical structure, it only maps onto a particular metrical level in this study. As expected, this effect was found in most conditions, thus suggesting that differences in the shape of the automatic response to auditory stimuli between older and younger adults are likely to explain the differences observed here. This result could also be consistent with the compensation hypothesis of aging, where older adults might increase activity to each single acoustic onset in order to compensate for hearing loss. The relative increase in amplitude at faster vs. slower frequencies observed here in older adults compared to younger adults is the opposite pattern to existing literature (e.g., Clinard et al., 2010; Goossens et al., 2016) and is surprising, given that perceiving and tracking beat subdivisions is more difficult than tracking a beat at fast tempi. One exception is F1 in the slow non-syncopated and syncopated conditions, where older adults have larger amplitudes than younger adults. Here, older adults show enhancement of the whole cycle rate, which may be a different form of compensation. It may also explain that older adults show higher averaged amplitudes of meter-related frequencies than younger adults at slow tempi. Further research is necessary to confirm and investigate these age effects at high and low frequencies in more detail.

Our spatial analysis, however, does not support a compensation theory of aging. In previous literature (Reuter-Lorenz and Cappell, 2008; Halpern et al., 2017), older adults demonstrate activity over a larger surface area in the frontal regions of the brain than younger adults. This pattern is not observed here, suggesting that older adults do not recruit larger neural areas to perform the same task as younger adults overall. Finally, the hypothesized interaction between age and rhythm, where older adults’ long-term exposure to Western music may help them compensate for the lack of prominent acoustic feature at meter-related frequencies in the syncopated rhythm was not borne out in our results, suggesting that such experience did not manifest itself in this particular study.

Importantly, the design with fixed order of conditions does not appear to have had a significant effect on our results. If fatigue had been an important factor, we would expect overall neural activity to decrease from one condition to the next. This is not the case, as illustrated by Figures 3, 4, which both present the data in the same order as participants completed blocks, from left to right. However, it is impossible to parse out any potential effects of fatigue. Though this is a disadvantage of a fixed order design, it does have an advantage. Due to the endogenous nature of meter perception, one way to capture indices of the perceived meter is to ask the participants to tap periodically while listening to the rhythm. The participants of this study could successfully perform this task as indicated by the obtained mean and standard deviation of the inter-tap intervals (see Supplementary material 2 for details), though the tapping sequences were too short to further compare tapping performance across conditions. A fixed order ensures that each participant has received the same amount of “practice” before each condition and therefore has the same opportunity to learn to mentally establish the meter in each condition, which was identified as important in pilot participants. Conversely, while a counterbalanced order design would eliminate any order or fatigue effects, meter perception in each condition may have differed due to more or less experience with the stimuli over time, depending on where the condition occurred in the experiment and was expected to be especially difficult if the fast, syncopated condition was presented first. That being said, the fixed presentation order of the stimuli is indeed a confound, since the neural enhancement to metrical frequencies could be related to the previous non-syncopated condition at the same tempo and the preceding tapping periods involving motor learning of the meter. Future work should explore a counterbalanced design to (i) ensure that the effects reported here are not related to fatigue and (ii) exploit this potential learning effect from the order of the blocks to directly test the link between enhanced neural activity at meter frequencies and the ability to tap the perceived meter. Finally, though tapping data was not the focus of this study, tapping did converge on a tapping rate corresponding to groupings of four events, in line with previous behavioral work (Lenc et al., 2018; Nozaradan et al., 2018a). The impact of aging on sensori-motor synchronization is an important question, and whether tapping on these rhythms is more variable in older participants remains to be investigated with specific designs.

To summarize, this study has demonstrated subtle differences between older and younger adults on neural activity to a rhythmic stimulus. In older adults, we observed preserved selective enhancement of neural activity at meter-related frequencies despite significant hearing loss, even in the syncopated rhythms in which the meter-related frequencies were not prominent acoustic features in the input. Moreover, younger adults showed lower amplitude at the frequency corresponding to single acoustic events making up the sequences relative to the other, slower meter-related frequencies, which were proportionally larger. In contrast, this difference was reduced in older adults, who showed relatively larger responses at the frequency of single acoustic events compared to activity elicited at slower frequencies. These results are compatible with previous results showing increased amplitude of N1-P2 complexes in response to single acoustic events and indicate that this effect of aging can also affect responses to acoustic events presented in long rhythmic sequences. Future research is necessary to better understand the difference in response shape between younger and older adults.

## Data availability statement

The raw data supporting the conclusions of this article will be made available by the authors, without undue reservation.

## Ethics statement

The studies involving human participants were reviewed and approved by Interdisciplinary Committee on Ethics in Human Research at Memorial University of Newfoundland. The patients/participants provided their written informed consent to participate in this study.

## Author contributions

SS: formal analysis and writing—original draft, review, and editing. EB: investigation and formal analysis. SN: conceptualization, methodology, and writing—review and editing. BZ: conceptualization, funding acquisition, supervision, resources, and writing—review and editing. All authors contributed to the article and approved the submitted version.

## Footnotes

1
https://www.letswave.org/
