# Supplementary material for: Aging effects on neural processing of rhythm and meter
Source: Front Aging Neurosci. 2022 Sep 1;14:848608. doi: 10.3389/fnagi.2022.848608 (PMC9475293; doi:10.3389/fnagi.2022.848608)
Supplement: Supplementary file 1 [file Data_Sheet_1.docx]

## Supplementary Materials 1 - Summary statistics, model specification details and follow-up t-test results

## Table 1. Summary statistics for meter-unrelated peaks

| Age | Tempo | Rhythm | Frequency | MeanAmplitude | StandardError |
| --- | --- | --- | --- | --- | --- |
| Younger | Slow | Non-syncopated | 0.83 | 0.0780057 | 0.0084729 |
| Younger | Slow | Non-syncopated | 1.66 | 0.0142428 | 0.0030411 |
| Younger | Slow | Non-syncopated | 2.08 | 0.0203644 | 0.0032852 |
| Younger | Slow | Non-syncopated | 2.91 | 0.0375499 | 0.0039400 |
| Younger | Slow | Non-syncopated | 3.33 | 0.0347361 | 0.0026435 |
| Younger | Slow | Non-syncopated | 3.75 | 0.0307553 | 0.0028445 |
| Younger | Slow | Non-syncopated | 4.16 | 0.0082533 | 0.0019121 |
| Younger | Slow | Non-syncopated | 4.57 | 0.0000824 | 0.0015144 |
| Younger | Slow | Syncopated | 0.83 | 0.0684450 | 0.0054068 |
| Younger | Slow | Syncopated | 1.66 | 0.0651119 | 0.0036049 |
| Younger | Slow | Syncopated | 2.08 | 0.0422580 | 0.0032515 |
| Younger | Slow | Syncopated | 2.91 | 0.0286044 | 0.0021018 |
| Younger | Slow | Syncopated | 3.33 | 0.0088772 | 0.0015495 |
| Younger | Slow | Syncopated | 3.75 | 0.0203156 | 0.0024042 |
| Younger | Slow | Syncopated | 4.16 | 0.0223526 | 0.0026422 |
| Younger | Slow | Syncopated | 4.57 | 0.0026887 | 0.0014577 |
| Younger | Fast | Non-syncopated | 1.66 | 0.0192275 | 0.0029082 |
| Younger | Fast | Non-syncopated | 3.33 | 0.0231274 | 0.0022974 |
| Younger | Fast | Non-syncopated | 4.16 | 0.0385591 | 0.0024789 |
| Younger | Fast | Non-syncopated | 5.83 | 0.0149206 | 0.0023665 |
| Younger | Fast | Non-syncopated | 6.66 | 0.0202689 | 0.0019773 |
| Younger | Fast | Non-syncopated | 7.50 | 0.0382612 | 0.0037386 |
| Younger | Fast | Non-syncopated | 8.33 | 0.0101402 | 0.0013623 |
| Younger | Fast | Non-syncopated | 9.16 | 0.0004522 | 0.0010637 |
| Younger | Fast | Syncopated | 1.66 | 0.0372886 | 0.0064486 |
| Younger | Fast | Syncopated | 3.33 | 0.0857562 | 0.0042886 |
| Younger | Fast | Syncopated | 4.16 | 0.0579578 | 0.0039490 |
| Younger | Fast | Syncopated | 5.83 | 0.0258373 | 0.0033213 |
| Younger | Fast | Syncopated | 6.66 | 0.0099667 | 0.0022307 |
| Younger | Fast | Syncopated | 7.50 | 0.0095987 | 0.0022955 |
| Younger | Fast | Syncopated | 8.33 | 0.0052247 | 0.0019539 |
| Younger | Fast | Syncopated | 9.16 | 0.0143075 | 0.0013541 |
| Older | Slow | Non-syncopated | 0.83 | 0.0159153 | 0.0068904 |
| Older | Slow | Non-syncopated | 1.66 | 0.0231684 | 0.0033219 |
| Older | Slow | Non-syncopated | 2.08 | 0.0068296 | 0.0024809 |
| Older | Slow | Non-syncopated | 2.91 | 0.0058791 | 0.0021596 |
| Older | Slow | Non-syncopated | 3.33 | 0.0148497 | 0.0025553 |
| Older | Slow | Non-syncopated | 3.75 | 0.0431644 | 0.0035857 |
| Older | Slow | Non-syncopated | 4.16 | 0.0011534 | 0.0018447 |
| Older | Slow | Non-syncopated | 4.57 | 0.0052663 | 0.0014733 |
| Older | Slow | Syncopated | 0.83 | 0.0526863 | 0.0049276 |
| Older | Slow | Syncopated | 1.66 | 0.0295822 | 0.0015906 |
| Older | Slow | Syncopated | 2.08 | 0.0279113 | 0.0017079 |
| Older | Slow | Syncopated | 2.91 | 0.0206890 | 0.0015823 |
| Older | Slow | Syncopated | 3.33 | 0.0157329 | 0.0012585 |
| Older | Slow | Syncopated | 3.75 | 0.0269940 | 0.0016075 |
| Older | Slow | Syncopated | 4.16 | 0.0114314 | 0.0011923 |
| Older | Slow | Syncopated | 4.57 | 0.0082055 | 0.0006783 |
| Older | Fast | Non-syncopated | 1.66 | 0.0286830 | 0.0036746 |
| Older | Fast | Non-syncopated | 3.33 | 0.0266107 | 0.0022738 |
| Older | Fast | Non-syncopated | 4.16 | 0.0204777 | 0.0018767 |
| Older | Fast | Non-syncopated | 5.83 | 0.0086279 | 0.0016437 |
| Older | Fast | Non-syncopated | 6.66 | 0.0147253 | 0.0015365 |
| Older | Fast | Non-syncopated | 7.50 | 0.0417021 | 0.0025352 |
| Older | Fast | Non-syncopated | 8.33 | 0.0070732 | 0.0010811 |
| Older | Fast | Non-syncopated | 9.16 | 0.0073590 | 0.0010355 |
| Older | Fast | Syncopated | 1.66 | 0.0500489 | 0.0082244 |
| Older | Fast | Syncopated | 3.33 | 0.0528557 | 0.0039821 |
| Older | Fast | Syncopated | 4.16 | 0.0538440 | 0.0033813 |
| Older | Fast | Syncopated | 5.83 | 0.0141220 | 0.0020992 |
| Older | Fast | Syncopated | 6.66 | 0.0096782 | 0.0012665 |
| Older | Fast | Syncopated | 7.50 | 0.0163796 | 0.0019675 |
| Older | Fast | Syncopated | 8.33 | 0.0200754 | 0.0016987 |
| Older | Fast | Syncopated | 9.16 | 0.0096550 | 0.0016027 |

## Table 2. Summary statistics for meter-related peaks

| Age | Tempo | Rhythm | Frequency | MeanAmplitude | StandardError |
| --- | --- | --- | --- | --- | --- |
| Younger | Slow | Non-syncopated | 0.41 | -0.0090419 | 0.0116278 |
| Younger | Slow | Non-syncopated | 1.25 | 0.2139752 | 0.0086961 |
| Younger | Slow | Non-syncopated | 2.50 | 0.0599340 | 0.0043373 |
| Younger | Slow | Non-syncopated | 5.00 | 0.0833212 | 0.0068019 |
| Younger | Slow | Syncopated | 0.41 | 0.0492761 | 0.0101707 |
| Younger | Slow | Syncopated | 1.25 | 0.1043801 | 0.0056635 |
| Younger | Slow | Syncopated | 2.50 | 0.0147049 | 0.0024240 |
| Younger | Slow | Syncopated | 5.00 | 0.0719328 | 0.0065555 |
| Younger | Fast | Non-syncopated | 0.83 | 0.0672375 | 0.0055117 |
| Younger | Fast | Non-syncopated | 2.50 | 0.1869922 | 0.0083230 |
| Younger | Fast | Non-syncopated | 5.00 | 0.0410084 | 0.0027795 |
| Younger | Fast | Non-syncopated | 10.00 | 0.0884871 | 0.0051882 |
| Younger | Fast | Syncopated | 0.83 | 0.0745292 | 0.0100574 |
| Younger | Fast | Syncopated | 2.50 | 0.1687050 | 0.0069872 |
| Younger | Fast | Syncopated | 5.00 | 0.0232339 | 0.0023889 |
| Younger | Fast | Syncopated | 10.00 | 0.0799961 | 0.0050560 |
| Older | Slow | Non-syncopated | 0.41 | -0.0366809 | 0.0137984 |
| Older | Slow | Non-syncopated | 1.25 | 0.1300885 | 0.0066984 |
| Older | Slow | Non-syncopated | 2.50 | 0.0342812 | 0.0036252 |
| Older | Slow | Non-syncopated | 5.00 | 0.1233665 | 0.0048650 |
| Older | Slow | Syncopated | 0.41 | 0.0886093 | 0.0074337 |
| Older | Slow | Syncopated | 1.25 | 0.0747768 | 0.0033805 |
| Older | Slow | Syncopated | 2.50 | 0.0145671 | 0.0015539 |
| Older | Slow | Syncopated | 5.00 | 0.0754394 | 0.0035759 |
| Older | Fast | Non-syncopated | 0.83 | 0.0286081 | 0.0059339 |
| Older | Fast | Non-syncopated | 2.50 | 0.1709451 | 0.0117460 |
| Older | Fast | Non-syncopated | 5.00 | 0.0308389 | 0.0021506 |
| Older | Fast | Non-syncopated | 10.00 | 0.1190852 | 0.0058380 |
| Older | Fast | Syncopated | 0.83 | 0.0185427 | 0.0067978 |
| Older | Fast | Syncopated | 2.50 | 0.1005168 | 0.0048617 |
| Older | Fast | Syncopated | 5.00 | 0.0041525 | 0.0011969 |
| Older | Fast | Syncopated | 10.00 | 0.1102473 | 0.0049182 |

## Table 3. Summary of the maximally fitted multiple linear regression model predicting spectral amplitude for meter-related and meter-unrelated peak frequencies including coefficient, Wald 95% confidence intervals and R^2^ for each predictor (where there are multiple levels, R^2^ is given for the predictor as a whole). Random effects account for < 0.00 variance and are therefore not reported.

| Predictor | Coefficient | 2.5% | 98.5% | R^2^ |
| --- | --- | --- | --- | --- |
| Intercept | 0.089 | 0.078 | 0.101 | 0.067 |
| FrequencyType | -0.059 | -0.065 | -0.053 | 0.100 |
| Rhythm | -0.027 | -0.037 | -0.017 | 0.001 |
| Tempo | 0.009 | 0.001 | 0.017 | 0.000 |
| Age | -0.021 | -0.038 | -0.004 | -0.000 |
| PTA | -0.000 | -0.001 | 0.000 | 0.000 |
| FrequencyType:Rhythm | 0.031 | 0.022 | 0.040 | 0.006 |
| FrequencyType:Tempo | -0.016 | -0.025 | -0.007 | 0.002 |
| Rhythm:Tempo | 0.018 | 0.007 | 0.028 | 0.000 |
| FrequencyType:Age | 0.011 | 0.002 | 0.020 | 0.001 |
| Rhythm:Age | 0.028 | 0.013 | 0.042 | 0.000 |
| Tempo:Age | 0.016 | 0.004 | 0.027 | 0.000 |
| FrequencyType:Rhythm:Tempo | -0.012 | -0.024 | 0.001 | 0.000 |
| FrequencyType:Rhythm:Age | -0.022 | -0.035 | -0.010 | 0.000 |
| FrequencyType:Tempo:Age | -0.003 | -0.016 | 0.009 | 0.001 |
| Rhythm:Tempo:Age | -0.047 | -0.062 | -0.033 | 0.001 |
| FrequencyType:Rhythm:Tempo:Age | 0.041 | 0.023 | 0.058 | 0.001 |

## Table 4. Follow-up t-test results comparing age groups for meter-related and meter-unrelated frequencies in each condition including t-statistic, p-value and Cohen’s d effect size.

| Tempo | Rhythm | FrequencyType | t-statistic | p-value | Effect size |
| --- | --- | --- | --- | --- | --- |
| Slow | Non-syncopated | Related | 3.421 | 0.001 | 0.183 |
| Slow | Non-syncopated | Unrelated | 6.776 | 0.000 | 0.256 |
| Slow | Syncopated | Related | -0.745 | 0.456 | -0.040 |
| Slow | Syncopated | Unrelated | 5.440 | 0.000 | 0.208 |
| Fast | Non-syncopated | Related | 1.533 | 0.125 | 0.082 |
| Fast | Non-syncopated | Unrelated | 0.995 | 0.320 | 0.038 |
| Fast | Syncopated | Related | 5.716 | 0.000 | 0.305 |
| Fast | Syncopated | Unrelated | 1.193 | 0.233 | 0.045 |

## Table 5. Summary of maximally fitted multiple linear regression model predicting spectral amplitude at meter-related frequency peaks, including coefficient, Wald 95% confidence intervals and R^2^ for each predictor (where there are multiple levels, R^2^ is given for the predictor as a whole). Random effects account for < 0.00 variance and coefficients for all channel predictors are < 0.00, and are therefore not reported.

| Predictor | Coefficient | 2.5% | 98.5% | R^2^ |
| --- | --- | --- | --- | --- |
| (Intercept) | 0.215 | 0.189 | 0.241 | 0.126 |
| Age | -0.078 | -0.114 | -0.042 | 0.000 |
| Tempo | -0.027 | -0.045 | -0.009 | 0.009 |
| Rhythm | -0.110 | -0.131 | -0.088 | -0.000 |
| F1 | -0.223 | -0.240 | -0.206 | 0.201 |
| F6 | -0.154 | -0.171 | -0.137 |  |
| F12 | -0.131 | -0.148 | -0.113 |  |
| PTA | 0.002 | -0.009 | 0.012 | 0.000 |
| Age:Tempo | 0.068 | 0.042 | 0.093 | 0.000 |
| Age:Rhythm | 0.054 | 0.025 | 0.083 | 0.000 |
| Tempo:Rhythm | 0.091 | 0.067 | 0.116 | 0.000 |
| Age:F1 | 0.056 | 0.032 | 0.080 | 0.016 |
| Age:F6 | 0.058 | 0.034 | 0.082 |  |
| Age:F12 | 0.124 | 0.100 | 0.148 |  |
| Tempo:F1 | 0.103 | 0.079 | 0.128 | 0.004 |
| Tempo:F6 | 0.008 | -0.016 | 0.032 |  |
| Tempo:F12 | 0.032 | 0.008 | 0.056 |  |
| Rhythm:F1 | 0.168 | 0.144 | 0.192 | 0.035 |
| Rhythm:F6 | 0.064 | 0.040 | 0.089 |  |
| Rhythm:F12 | 0.098 | 0.074 | 0.123 |  |
| Age:Tempo:Rhythm | -0.106 | -0.140 | -0.073 | 0.003 |
| Age:Tempo:F1 | -0.079 | -0.113 | -0.045 | 0.004 |
| Age:Tempo:F6 | -0.052 | -0.086 | -0.019 |  |
| Age:Tempo:F12 | -0.077 | -0.111 | -0.043 |  |
| Age:Rhythm:F1 | 0.013 | -0.021 | 0.047 | 0.001 |
| Age:Rhythm:F6 | -0.029 | -0.063 | 0.005 |  |
| Age:Rhythm:F12 | -0.091 | -0.125 | -0.057 |  |
| Tempo:Rhythm:F1 | -0.142 | -0.177 | -0.108 | 0.015 |
| Tempo:Rhythm:F6 | -0.064 | -0.098 | -0.029 |  |
| Tempo:Rhythm:F12 | -0.088 | -0.123 | -0.054 |  |
| Age:Tempo:Rhythm:F1 | 0.022 | -0.026 | 0.070 | 0.004 |
| Age:Tempo:Rhythm:F6 | 0.072 | 0.024 | 0.120 |  |
| Age:Tempo:Rhythm:F12 | 0.143 | 0.095 | 0.190 |  |

## Table 6. Follow-up t-test results for neural activity amplitudes at meter-related frequencies comparing age groups at each peak and condition including t-statistic, p-value and Cohen’s d effect size.

| Peak | Condition | t-statistic | p-value | Effect size |
| --- | --- | --- | --- | --- |
| F3 | Non-syncopated - Slow | 1.532 | 0.127 | 0.163 |
| F1 | Non-syncopated - Slow | 7.642 | 0.000 | 0.764 |
| F6 | Non-syncopated - Slow | 4.538 | 0.000 | 0.476 |
| F12 | Non-syncopated - Slow | -4.789 | 0.000 | -0.503 |
| F3 | Syncopated - Slow | -3.122 | 0.002 | -0.334 |
| F1 | Syncopated - Slow | 4.488 | 0.000 | 0.476 |
| F6 | Syncopated - Slow | 0.048 | 0.962 | 0.005 |
| F12 | Syncopated - Slow | -0.470 | 0.639 | -0.051 |
| F3 | Non-syncopated - Fast | 4.770 | 0.000 | 0.495 |
| F1 | Non-syncopated - Fast | 1.115 | 0.266 | 0.118 |
| F6 | Non-syncopated - Fast | 2.894 | 0.004 | 0.309 |
| F12 | Non-syncopated - Fast | -3.918 | 0.000 | -0.410 |
| F3 | Syncopated - Fast | 4.612 | 0.000 | 0.486 |
| F1 | Syncopated - Fast | 8.011 | 0.000 | 0.798 |
| F6 | Syncopated - Fast | 7.141 | 0.000 | 0.729 |
| F12 | Syncopated - Fast | -4.289 | 0.000 | -0.449 |

## Table 7. Follow-up t-test results for meter-related peak frequencies comparing F3 and F1 for each age group and condition including t-statistic, p-value and Cohen’s d effect size.

| Age | Condition | t-statistic | p-value | Effect size |
| --- | --- | --- | --- | --- |
| Younger | Non-syncopated - Slow | -15.359 | 0.000 | -1.285 |
| Younger | Syncopated - Slow | -4.734 | 0.000 | -0.501 |
| Older | Non-syncopated - Slow | -10.873 | 0.000 | -0.995 |
| Older | Syncopated - Slow | 1.694 | 0.092 | 0.178 |
| Younger | Non-syncopated - Fast | -11.996 | 0.000 | -1.096 |
| Younger | Syncopated - Fast | -7.690 | 0.000 | -0.775 |
| Older | Non-syncopated - Fast | -10.816 | 0.000 | -0.991 |
| Older | Syncopated - Fast | -9.809 | 0.000 | -0.919 |

## Table 8. Follow-up t-test results for meter-related peak frequencies comparing F3 and F12 for each age group and condition including t-statistic, p-value and Cohen’s d effect size.

| Age | Condition | t-statistic | p-value | Effect size |
| --- | --- | --- | --- | --- |
| Younger | Non-syncopated - Slow | 11.834 | 0.000 | 1.085 |
| Younger | Syncopated - Slow | 3.745 | 0.000 | 0.401 |
| Older | Non-syncopated - Slow | 0.812 | 0.417 | 0.086 |
| Older | Syncopated - Slow | -0.135 | 0.893 | -0.014 |
| Younger | Non-syncopated - Fast | 10.044 | 0.000 | 0.962 |
| Younger | Syncopated - Fast | 10.286 | 0.000 | 0.979 |
| Older | Non-syncopated - Fast | 3.954 | 0.000 | 0.409 |
| Older | Syncopated - Fast | -1.407 | 0.160 | -0.148 |

## Table 9. Summary of maximally fitted multiple linear regression model predicting spectral amplitude at meter-related frequency peaks over multiple electrode montages, including coefficient, Wald 95% confidence intervals and R^2^ for each predictor (where there are multiple levels, R^2^ is given for the predictor as a whole). Random effects and coefficients for all channel predictors account for < 0.00 of model variation, and are therefore not reported.

| Predictor | Coefficient | 2.5% | 98.5% | R^2^ |
| --- | --- | --- | --- | --- |
| (Intercept) | 0.219 | 0.183 | 0.256 | 0.0337973 |
| Age | -0.075 | -0.127 | -0.024 | 0.0000035 |
| Tempo | -0.027 | -0.067 | 0.013 | 0.0022733 |
| Rhythm | -0.110 | -0.151 | -0.068 | 0.0000095 |
| F1 | -0.223 | -0.262 | -0.184 | 0.0445038 |
| F6 | -0.154 | -0.193 | -0.115 |  |
| F12 | -0.131 | -0.170 | -0.091 |  |
| Ring1 | -0.020 | -0.060 | 0.020 | 0.0041681 |
| Ring2 | -0.055 | -0.094 | -0.017 |  |
| Ring3 | -0.078 | -0.117 | -0.040 |  |
| PTA | -0.001 | -0.002 | 0.001 | -0.0000014 |
| Age:Tempo | 0.068 | 0.012 | 0.123 | 0.0000103 |
| Age:Rhythm | 0.054 | -0.003 | 0.112 | 0.0000131 |
| Tempo:Rhythm | 0.091 | 0.036 | 0.147 | 0.0009881 |
| Age:F1 | 0.056 | 0.002 | 0.111 | 0.0016876 |
| Age:F6 | 0.058 | 0.004 | 0.113 |  |
| Age:F12 | 0.124 | 0.069 | 0.179 |  |
| Tempo:F1 | 0.103 | 0.048 | 0.159 | 0.0035917 |
| Tempo:F6 | 0.008 | -0.047 | 0.064 |  |
| Tempo:F12 | 0.032 | -0.023 | 0.088 |  |
| Rhythm:F1 | 0.168 | 0.112 | 0.223 | 0.0109811 |
| Rhythm:F6 | 0.064 | 0.009 | 0.120 |  |
| Rhythm:F12 | 0.098 | 0.043 | 0.154 |  |
| Age:Ring1 | 0.000 | -0.055 | 0.055 | 0.0000328 |
| Age:Ring2 | 0.031 | -0.022 | 0.085 |  |
| Age:Ring3 | 0.027 | -0.026 | 0.081 |  |
| Tempo:Ring1 | -0.006 | -0.062 | 0.051 | 0.0000275 |
| Tempo:Ring2 | -0.008 | -0.063 | 0.046 |  |
| Tempo:Ring3 | -0.005 | -0.060 | 0.049 |  |
| Rhythm:Ring1 | 0.009 | -0.048 | 0.066 | 0.0002645 |
| Rhythm:Ring2 | 0.034 | -0.020 | 0.089 |  |
| Rhythm:Ring3 | 0.046 | -0.009 | 0.100 |  |
| F1:Ring1 | 0.026 | -0.031 | 0.082 | 0.0010766 |
| F6:Ring1 | 0.007 | -0.050 | 0.064 |  |
| F12:Ring1 | 0.018 | -0.039 | 0.075 |  |
| F1:Ring2 | 0.048 | -0.007 | 0.102 |  |
| F6:Ring2 | 0.033 | -0.021 | 0.088 |  |
| F12:Ring2 | 0.041 | -0.013 | 0.096 |  |
| F1:Ring3 | 0.049 | -0.005 | 0.104 |  |
| F6:Ring3 | 0.049 | -0.005 | 0.104 |  |
| F12:Ring3 | 0.061 | 0.006 | 0.115 |  |
| Age:Tempo:Rhythm | -0.106 | -0.184 | -0.029 | 0.0030460 |
| Age:Tempo:F1 | -0.079 | -0.156 | -0.002 | 0.0013103 |
| Age:Tempo:F6 | -0.052 | -0.130 | 0.025 |  |
| Age:Tempo:F12 | -0.077 | -0.154 | -0.000 |  |
| Age:Rhythm:F1 | 0.013 | -0.064 | 0.090 | 0.0013769 |
| Age:Rhythm:F6 | -0.029 | -0.106 | 0.048 |  |
| Age:Rhythm:F12 | -0.091 | -0.168 | -0.014 |  |
| Tempo:Rhythm:F1 | -0.142 | -0.221 | -0.064 | 0.0041504 |
| Tempo:Rhythm:F6 | -0.064 | -0.142 | 0.015 |  |
| Tempo:Rhythm:F12 | -0.088 | -0.167 | -0.010 |  |
| Age:Tempo:Ring1 | 0.015 | -0.063 | 0.093 | 0.0000678 |
| Age:Tempo:Ring2 | 0.052 | -0.024 | 0.127 |  |
| Age:Tempo:Ring3 | 0.030 | -0.045 | 0.105 |  |
| Age:Rhythm:Ring1 | 0.002 | -0.076 | 0.080 | 0.0000404 |
| Age:Rhythm:Ring2 | -0.020 | -0.095 | 0.055 |  |
| Age:Rhythm:Ring3 | -0.017 | -0.092 | 0.058 |  |
| Tempo:Rhythm:Ring1 | -0.006 | -0.086 | 0.074 | 0.0003245 |
| Tempo:Rhythm:Ring2 | -0.026 | -0.103 | 0.051 |  |
| Tempo:Rhythm:Ring3 | -0.048 | -0.125 | 0.029 |  |
| Age:F1:Ring1 | -0.029 | -0.107 | 0.049 | 0.0006845 |
| Age:F6:Ring1 | 0.008 | -0.070 | 0.086 |  |
| Age:F12:Ring1 | -0.019 | -0.097 | 0.059 |  |
| Age:F1:Ring2 | -0.155 | -0.230 | -0.080 |  |
| Age:F6:Ring2 | -0.025 | -0.100 | 0.050 |  |
| Age:F12:Ring2 | -0.057 | -0.132 | 0.018 |  |
| Age:F1:Ring3 | -0.113 | -0.188 | -0.038 |  |
| Age:F6:Ring3 | -0.013 | -0.088 | 0.062 |  |
| Age:F12:Ring3 | -0.072 | -0.147 | 0.003 |  |
| Tempo:F1:Ring1 | -0.010 | -0.090 | 0.070 | 0.0008100 |
| Tempo:F6:Ring1 | 0.013 | -0.067 | 0.093 |  |
| Tempo:F12:Ring1 | -0.005 | -0.085 | 0.075 |  |
| Tempo:F1:Ring2 | -0.008 | -0.085 | 0.069 |  |
| Tempo:F6:Ring2 | 0.020 | -0.057 | 0.097 |  |
| Tempo:F12:Ring2 | -0.005 | -0.082 | 0.072 |  |
| Tempo:F1:Ring3 | 0.008 | -0.069 | 0.085 |  |
| Tempo:F6:Ring3 | 0.018 | -0.059 | 0.095 |  |
| Tempo:F12:Ring3 | -0.016 | -0.093 | 0.061 |  |
| Rhythm:F1:Ring1 | -0.045 | -0.125 | 0.035 | 0.0002221 |
| Rhythm:F6:Ring1 | 0.010 | -0.070 | 0.090 |  |
| Rhythm:F12:Ring1 | -0.011 | -0.091 | 0.069 |  |
| Rhythm:F1:Ring2 | -0.081 | -0.158 | -0.004 |  |
| Rhythm:F6:Ring2 | -0.008 | -0.085 | 0.069 |  |
| Rhythm:F12:Ring2 | -0.021 | -0.098 | 0.056 |  |
| Rhythm:F1:Ring3 | -0.116 | -0.193 | -0.039 |  |
| Rhythm:F6:Ring3 | -0.006 | -0.083 | 0.071 |  |
| Rhythm:F12:Ring3 | -0.036 | -0.113 | 0.041 |  |
| Age:Tempo:Rhythm:F1 | 0.022 | -0.087 | 0.131 | 0.0038060 |
| Age:Tempo:Rhythm:F6 | 0.072 | -0.037 | 0.181 |  |
| Age:Tempo:Rhythm:F12 | 0.143 | 0.033 | 0.252 |  |
| Age:Tempo:Rhythm:Ring1 | -0.002 | -0.112 | 0.108 |  |
| Age:Tempo:Rhythm:Ring2 | -0.041 | -0.147 | 0.066 |  |
| Age:Tempo:Rhythm:Ring3 | 0.012 | -0.094 | 0.118 |  |
| Age:Tempo:F1:Ring1 | 0.045 | -0.065 | 0.155 | 0.0001647 |
| Age:Tempo:F6:Ring1 | -0.020 | -0.131 | 0.090 |  |
| Age:Tempo:F12:Ring1 | -0.004 | -0.114 | 0.107 |  |
| Age:Tempo:F1:Ring2 | 0.094 | -0.012 | 0.200 |  |
| Age:Tempo:F6:Ring2 | -0.066 | -0.172 | 0.040 |  |
| Age:Tempo:F12:Ring2 | -0.038 | -0.144 | 0.068 |  |
| Age:Tempo:F1:Ring3 | 0.087 | -0.019 | 0.193 |  |
| Age:Tempo:F6:Ring3 | -0.027 | -0.133 | 0.079 |  |
| Age:Tempo:F12:Ring3 | -0.004 | -0.110 | 0.102 |  |
| Age:Rhythm:F1:Ring1 | 0.044 | -0.066 | 0.154 | 0.0004738 |
| Age:Rhythm:F6:Ring1 | -0.012 | -0.122 | 0.098 |  |
| Age:Rhythm:F12:Ring1 | 0.013 | -0.098 | 0.123 |  |
| Age:Rhythm:F1:Ring2 | 0.201 | 0.095 | 0.307 |  |
| Age:Rhythm:F6:Ring2 | 0.017 | -0.089 | 0.124 |  |
| Age:Rhythm:F12:Ring2 | 0.026 | -0.081 | 0.132 |  |
| Age:Rhythm:F1:Ring3 | 0.195 | 0.089 | 0.301 |  |
| Age:Rhythm:F6:Ring3 | 0.004 | -0.102 | 0.111 |  |
| Age:Rhythm:F12:Ring3 | 0.045 | -0.061 | 0.152 |  |
| Tempo:Rhythm:F1:Ring1 | 0.057 | -0.056 | 0.170 | 0.0001031 |
| Tempo:Rhythm:F6:Ring1 | -0.012 | -0.125 | 0.101 |  |
| Tempo:Rhythm:F12:Ring1 | 0.008 | -0.106 | 0.121 |  |
| Tempo:Rhythm:F1:Ring2 | 0.101 | -0.008 | 0.210 |  |
| Tempo:Rhythm:F6:Ring2 | 0.002 | -0.107 | 0.111 |  |
| Tempo:Rhythm:F12:Ring2 | 0.013 | -0.096 | 0.121 |  |
| Tempo:Rhythm:F1:Ring3 | 0.163 | 0.054 | 0.272 |  |
| Tempo:Rhythm:F6:Ring3 | 0.012 | -0.096 | 0.121 |  |
| Tempo:Rhythm:F12:Ring3 | 0.035 | -0.073 | 0.144 |  |
| Age:Tempo:Rhythm:F1:Ring1 | -0.079 | -0.235 | 0.077 | 0.0011288 |
| Age:Tempo:Rhythm:F6:Ring1 | 0.015 | -0.141 | 0.171 |  |
| Age:Tempo:Rhythm:F12:Ring1 | -0.010 | -0.166 | 0.146 |  |
| Age:Tempo:Rhythm:F1:Ring2 | -0.180 | -0.330 | -0.030 |  |
| Age:Tempo:Rhythm:F6:Ring2 | 0.056 | -0.095 | 0.206 |  |
| Age:Tempo:Rhythm:F12:Ring2 | 0.034 | -0.116 | 0.184 |  |
| Age:Tempo:Rhythm:F1:Ring3 | -0.264 | -0.414 | -0.114 |  |
| Age:Tempo:Rhythm:F6:Ring3 | -0.004 | -0.154 | 0.146 |  |
| Age:Tempo:Rhythm:F12:Ring3 | -0.037 | -0.187 | 0.113 |  |
