# Supplementary material for: Aging effects on neural processing of rhythm and meter
Source: Front Aging Neurosci. 2022 Sep 1;14:848608. doi: 10.3389/fnagi.2022.848608 (PMC9475293; doi:10.3389/fnagi.2022.848608)
Supplement: Supplementary file 2 [file Data_Sheet_2.docx]

**Supplementary Materials 2 – Tapping analysis**

In these supplementary materials, analysis of the tapping data is presented. Stimuli and procedure are as described in the main manuscript.

Mean inter-tap interval (ITI) was calculated for each participant and each condition and is plotted in Figure S1, where error bars represent standard deviation. Mean (standard deviation) ITIs for slow non-syncopated, fast non-syncopated, slow syncopated and fast syncopated were 781.1 (186.1), 400.34 (97.2), 791.4 (193.2) and 434.1 (135.7) respectively. A mixed effects multiple linear regression model predicting ITI with *tempo*, *rhythm* and *age* as fixed effects found a significant effect of tempo, but not of rhythm or age (see Table 1), nor are there any significant interactions between the predictors. More specifically, ITIs in response to fast stimuli were shorter than slow stimuli.

Together, Figure and Table 1 show that the majority of participants tapped at a metrical grouping of 4 events (800ms for the slow tempo and 400ms for the fast tempo). A few participants also tapped at a faster rate (grouping of 2 events), and some at a slower rate (grouping of 6 or 7 events).

**Table 1**. Summary of predictors from the maximally fitted multiple linear regression model predicting ITI including coefficient, Wald 95% confidence intervals and R^2^ for each predictor (where there are multiple levels, R^2^ is given for the predictor as a whole).

| **Predictor** | **Coefficient** | **2.5%** | **98.5%** | **R^2^** |
| --- | --- | --- | --- | --- |
| Intercept | 1464.3 | 838.6 | 2098.9 | - |
| Tempo | -530.3 | -829.9 | -230.7 | -.001 |
| Rhythm | -263.3 | -638.0 | 111.5 | .00 |
| Age | -198.4 | -594.9 | 198.1 | .00 |
| Tempo:Rhythm | 127.2 | -65.5 | 319.8 | .00 |
| Tempo:Age | 90.8 | -98.8 | 280.5 | .00 |
| Rhythm:Age | 175.0 | -60.3 | 410.2 | .00 |
| Tempo:Rhythm:Age | -74.5 | -195.0 | 46.0 | .00 |
| **Random effects** |  |  |  |  |
| Intercept | 21134 |  |  | .67 |
| Fast Nonsyncopated | 14652 |  |  |  |
| Slow Syncopated | 27363 |  |  |  |
| Fast Syncopated | 26364 |  |  |  |
| Residual | 19469 |  |  |  |
| 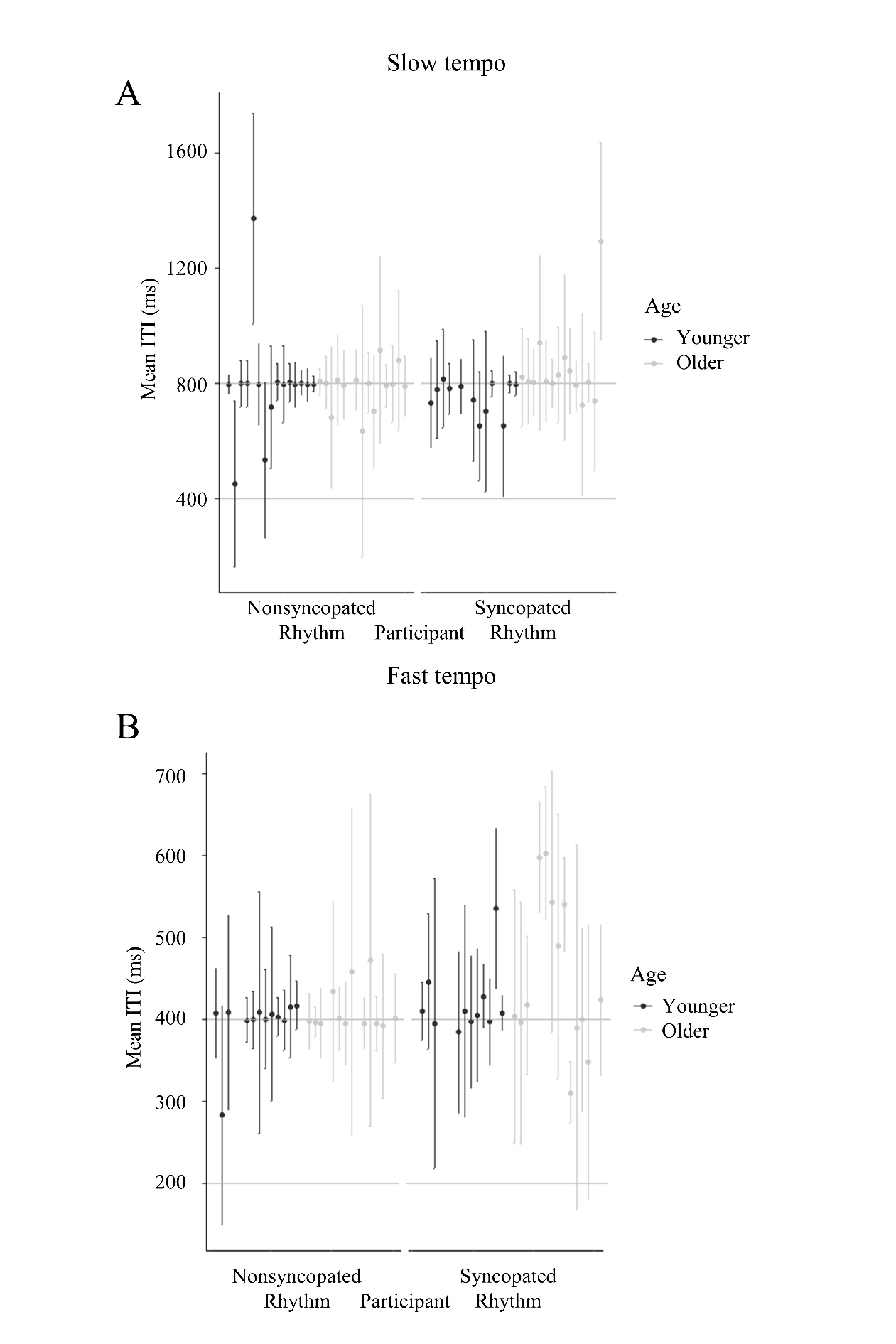 | | | | |
| **Figure S1**. Mean ITI for each participant for slow (A) and fast (B) tempi and non-syncopated and syncopated rhythms, divided by age group. Error bars represent standard deviation. | | | | |
